# Exploring the causal relationship between airborne particulate matter and ulcerative colitis: A two-sample mendelian randomization study

**DOI:** 10.1371/journal.pone.0300066

**Published:** 2024-03-08

**Authors:** Chong Fu, Qi Wang, Yan Chen, Yanping Zhang

**Affiliations:** Department of Gastroenterology, Anqing Municipal Hospital, Anqing, PR China; Kanazawa University, JAPAN

## Abstract

**Background:**

Existing research has demonstrated links between airborne particulate matter and ulcerative colitis (UC) onset. Through Mendelian randomization, this study aims to further delineate the causal association between specific types of airborne particulates and UC.

**Methods:**

A two-sample Mendelian randomization analysis was undertaken to investigate the causality between airborne particulate matter and UC. Genetic datasets for both airborne particulates and UC were derived from accessible genome-wide association studies (GWAS). We employed a range of MR techniques, such as inverse variance weighted (IVW), weighted median, MR-Egger, and Wald Ratio, to validate the causality. In addition, sensitivity assessments were executed to ensure result reliability.

**Results:**

The data indicate a probable positive correlation between PM_2.5_ exposure and UC risk (OR: 3.6; 95% CI: [1.2–11.3]; P = 0.026). The statistical strength for causal determination via the IVW approach stood at 0.87, with a Type I error rate set at 0.025. Assessments using Cochran’s Q test, MR-Egger intercept, MR-PRESSO, and leave-one-out sensitivity analyses did not identify notable heterogeneity, pleiotropy, or biases in the overall relationship between PM_2.5_ and UC. Furthermore, the MR-Steiger assessment indicated that PM_2.5_ exposure level determinants predominantly affect UC vulnerability.

**Conclusion:**

The findings underscore the potential involvement of PM_2.5_ in UC pathogenesis.

## 1. Introduction

Ulcerative colitis (UC) constitutes an inflammatory bowel disease predominantly affecting the rectocolon, manifesting symptoms including diarrhea, mucopurulent hematochezia, and abdominal discomfort. It also presents extraintestinal complications impacting the hepatobiliary system, skin, eyes, and musculoskeletal structures [1]. UC’s trajectory often involves chronic and recurrent episodes, with ongoing inflammatory stimuli occasionally leading to colorectal cancer development [2]. Reports indicate that UC afflicts approximately 150,000 individuals in Germany [3]. Despite rigorous investigation, understanding of UC’s exact etiology and pathogenesis remains substantially limited. Present theories propose that a complex interplay between environmental exposures, genetic factors, intestinal microbiota, and immunological responses may underlie the disease [4].

Air pollution, a widespread consequence of industrialization throughout the past century, has impacted nations in every global hemisphere. Its primary sources are combustion processes, including the burning of coal, oil, natural gas, and biomass fuels [5]. The World Health Organization reports that air pollution represents the foremost environmental risk worldwide, causing approximately seven million deaths in 2012 alone [6]. It significantly endangers human health and is intricately linked with various acute and chronic illnesses, including respiratory infections, cardiovascular diseases, and mental health disorders [7]. Notably, China experiences particularly acute air pollution challenges, exhibiting unique characteristics compared to developed regions such as the United States and Europe. As the leading global producer and consumer of coal, China’s challenges are compounded by outdated technologies and inadequate maintenance of coal-fired facilities, placing it at the forefront of both outdoor and indoor air pollution levels [8]. In environmental science, particulate matter refers to airborne solid particles or droplets, constituting a major source of air pollution. This matter is categorized by size into inhalable particulate matter (PM_10_), fine particles (PM_2.5_), and coarse particles (PM_2.5–10_). Fine particulate matter (PM_2.5_), with an aerodynamic diameter of 2.5 micrometers or less, remains in the atmosphere for prolonged periods and can infiltrate the bronchioles and alveoli, presenting significant health hazards. Epidemiological research has shown that exposure to environmental PM_2.5_ potentially aggravates autoimmune diseases, such as diabetes, rheumatoid arthritis, and multiple sclerosis [9, 10], and may also cause cytokine imbalances, leading to inflammatory reactions [11]. Consequently, amidst escalating global air pollution, comprehensive research into the effects of air pollutants on public health is of paramount public health and clinical relevance. Ananthakrishnan et al. conducted a study correlating hospitalization rates for UC in 72 Wisconsin counties with the annual average total emissions of environmental air pollutants, as monitored by the United States Environmental Protection Agency. This ecological analysis revealed a statistically significant correlation between increased UC hospitalizations and higher emissions of air gaseous pollutants [12]. Conversely, a European nested case-control study identified a negative correlation between fine particulate matter exposure and inflammatory bowel disease risk, not isolated to ulcerative colitis alone [13].

In examining the potential adverse effects of environmental factors on UC, especially the association with particulate matter, a notable research gap emerges. While some epidemiological data hint at a correlation, the inconsistency across study outcomes highlights the need for further investigation into the potential causal link between particulate matter exposure and UC. This research utilizes the Mendelian Randomization method to offer a more precise causal analysis than traditional observational studies, effectively reducing the influence of confounding variables. The ability to adjust particulate matter exposure to decrease UC’s incidence or recurrence is pivotal for shaping public health policies and refining clinical interventions. In clinical practice, understanding particulate matter’s role in the pathogenesis of UC is crucial for devising targeted treatment and prevention strategies for patients and those at risk in highly polluted areas. Additionally, raising public awareness about the connection between air pollution and UC plays a critical role in enhancing health literacy and facilitating the adoption of efficacious policies. Consequently, this investigation not only pursues scientific inquiry but also aims to contribute to public health enhancement and patient life quality improvement.

## 2. Methods

### 2.1 Study design

This study utilizes a two-sample Mendelian randomization approach to rigorously ascertain the causal association between airborne particulate matter and UC risk. The MR methodology is predicated on three core premises: first, that genetic instruments correlate directly with the exposure; second, that they are assumed not to correlate with the outcome and are autonomous of any confounders, known or otherwise; and third, that any effect of the instrumental variables on the outcome is exclusively mediated through the exposure under consideration. To avoid sample overlap, we sourced genetic data on airborne particulates and UC from distinct GWAS datasets. The MR analysis predominantly employed the IVW method to estimate the causality between airborne particulates and UC. This IVW approach yields reliable causal effect estimates, contingent upon the satisfaction of all three instrumental variable premises.

### 2.2 Data sources

The study designated airborne particulate matter, encompassing PM_2.5_, PM_2.5–10_, and PM_10_, as exposure entities, extracting all pertinent datasets from the UK Biobank. This comprehensive prospective study, comprising over half a million UK constituents, offers an array of published data spanning phenotypes, genetic details, and whole-genome genotyping. In the European cohort, the respective GWAS datasets for PM_2.5_ (GWAS ID: ukb-b-10817), PM_2.5–10_ (GWAS ID: ukb-b-12963), and PM_10_ (GWAS ID: ukb-b-589) encompass 423,796, 423,796, and 455,314 participants. Air particulate matter-related metrics were ascertained via Land Use Regression (LUR) models [14]. UC GWAS data were derived from a FinnGen consortium meta-analysis, incorporating the R9 release, with 2,251 UC instances and 210,300 healthy controls. This study entailed a secondary analysis of genome-wide association studies (GWAS) data, which is publicly accessible. Consequently, the requirement for additional ethical clearance was not applicable.

### 2.3 Selection of instrumental variables

To select instrumental variables (IVs) associated with airborne particulate matter, we applied specific criteria. The selection process involved a significance threshold of p < 5 × 10^−8^ and r^2^ < 0.001, with a proximity limit of 10,000 kilobases, targeting single nucleotide polymorphisms (SNPs) significantly correlated with the particulates. We computed the F-statistics for each SNP using predefined methods, excluding those with an F-statistic below 10 due to their classification as weak IVs [15].

### 2.4 Mendelian randomization analysis

We adopted five MR methods to investigate the causality between airborne particulate matter and UC, utilizing the IVW approach as the primary analysis, supported by MR-Egger, Weighted Median, and additional methods. We measured potential causal links with odds ratios (ORs). Heterogeneity was evaluated using Cochran’s Q statistic in both mr_egger and IVW methods, with p-values above 0.05 indicating homogeneity [16, 17]. We examined pleiotropy through the intercept from MR-Egger and global test p-values from MR-PRESSO, interpreting p > 0.05 as the absence of substantive pleiotropy [18, 19]. Additionally, we undertook "leave-one-out" sensitivity analyses to confirm the independence of exposure causality from individual SNPs. We classified associations as significant at p-values < 0.025 (adjusted for multiple comparisons) and as suggestive with p-values within 0.025 to 0.05. All analyses were two-sided, performed using the TwoSampleMR and MR-PRESSO packages in R (version 4.2.0).

### 2.5 Statistical power calculation

We assessed the statistical robustness of our MR analysis via an online calculator (https://sb452.shinyapps.io/power) [20]. This evaluation was based on parameters such as total sample size, a 0.025 significance level, the variance in exposure attributable to IVs (R^2^), and the case-control balance, targeting a minimum power threshold of 0.8 to confidently negate false null hypotheses [21].

## 3. Results

### 3.1. Causal effect of airborne particulate matter on UC

Employing a stringent threshold of 5 × 10^−8^, we identified 30 SNPs associated with two types of airborne pollutants. The F-statistics for the IVs were all above 10, indicating the absence of weak instrument bias and affirming the reliability of the results. The impact on UC at various SNP sites was determined through two-sample Mendelian randomization (S1 Table in S1 File).

**Fig 1:**
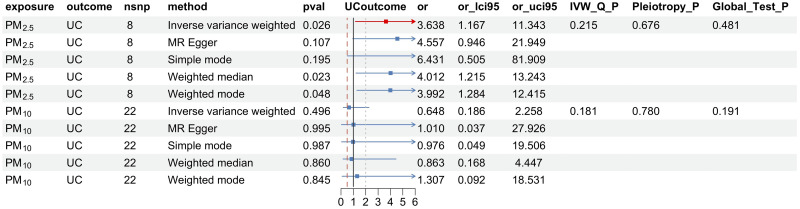
Causal correlations of airborne particulate matter on UC. NSNP: The final number of SNPs used in the analysis. OR: The estimated effect of airborne particulate matter on UC. IVW_Q_P: The P value of the Cochran Q test. Pleiotropy_P: The P value of the MR-Egger regression intercept hypothesis test. Global_Test_P: The P value of the MR-PRESSO global test.

**Fig 2:**
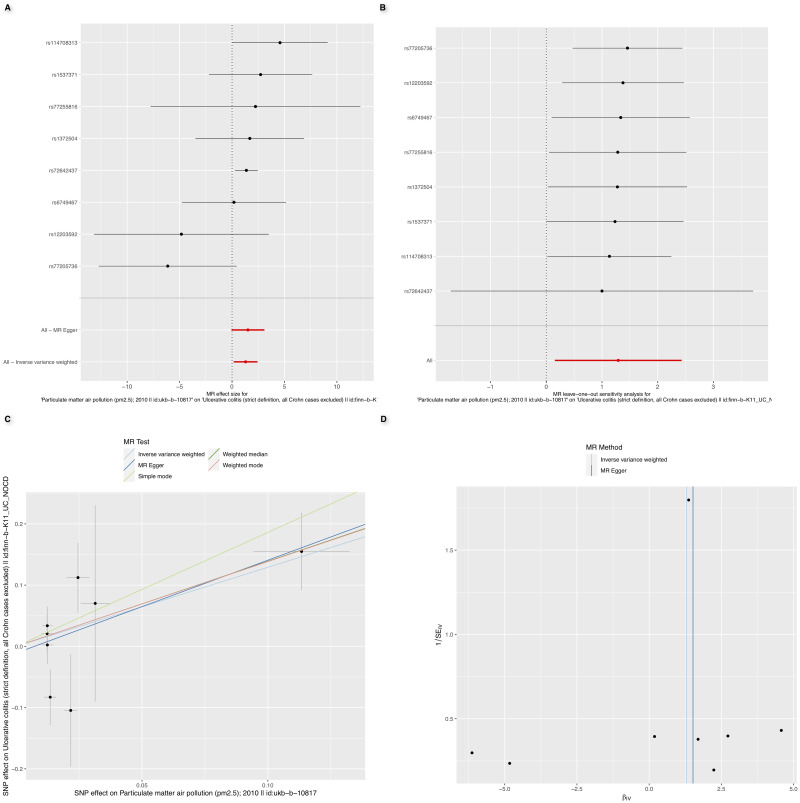
(A) Forest plot of the two—samples Mendelian Randomization analysis; (B) Result of “leave—one—out” sensitivity analysis; (C) Scatter plot of the two—samples Mendelian Randomization analysis; (D) Funnel plot of the two—sample Mendelian Randomization analysis.

As depicted in Fig 1 and S2 Table in S1 File, rigorous screening with a threshold of P<5×10^−8^ enabled us to discern two types of airborne particulates. Notably, a positive correlation was found between pm_2.5_ levels and UC risk (OR: 3.6; 95% CI: [1.2–11.3]; P = 0.026) (Fig 2A and 2C). Furthermore, causal inference obtained through the IVW method revealed a statistical power of 0.91 with a type I error rate of 0.05. Cochran’s Q test demonstrated no significant heterogeneity between overall PM_2.5_ levels and UC (P = 0.215). According to MR-Egger intercept and MR-PRESSO testing, no evidence supported the presence of directional pleiotropy, and the funnel plot similarly indicated no bias in the study (Fig 2D). Additionally, the MR-Steiger test was utilized to ascertain the direction of the causal effect, confirming that variations in PM_2.5_ levels influence UC susceptibility, not vice versa, with robust directional findings (S3 Table in S1 File). Moreover, ’leave-one-out’ sensitivity analyses were conducted to verify the influence of each SNP site on the overall causal relationship. Results indicated no significant changes in the aforementioned causal relationship when systematically removing a single SNP and repeating MR analysis (Fig 2B), proving that the estimated effect cannot be attributed to any individual genetic instrument. No causal relationship was observed between PM_10_ and UC.

Despite the p-value of 0.026, marginally exceeding the Bonferroni correction threshold of 0.025, the IVW method substantiates PM_2.5_’s causal implication in UC. The IVW p-value closely approaches the conservative Bonferroni standard, reinforcing the validity of PM_2.5_’s role. The consistency of findings from WM, MR-Egger, and Maximum Likelihood Estimation (MBE) further supports this, particularly with WM’s p-value aligning with the Bonferroni criterion. Moreover, the maintenance of a statistical power of 0.87 at a stringent alpha level of 0.025 highlights the study’s outcomes’ high reliability. Notably, removing PM_10_ from the analyses enables compliance with the Bonferroni threshold. However, a broad inclusion of particulate matter classifications for exposure consideration affords a macroscopic evaluation of airborne particulates’ cumulative effect on UC.

## 4. Discussion

In our study, we utilized the Mendelian randomization approach, primarily employing the IVW method, complemented by the WM method, to investigate the potential relationship between PM_2.5_ exposure and UC. While the p-value for the IVW method approached the threshold of statistical significance after multiple corrections, the WM method’s p-value was significantly lower, denoting statistical significance. Notably, the MR-Egger-intercept and MRPRESSO tests found no evidence of horizontal pleiotropy, enhancing the robustness of our findings. This indicates that although a single method might be affected by multiple corrections, a holistic approach using diverse methods offers a more thorough perspective. We recognize that while multiple corrections aid in controlling Type I errors, they can occasionally be excessively stringent, thereby possibly diminishing the likelihood of identifying true effects. Therefore, in interpreting our results, we took into account not only statistical significance but also power analysis and the real-world implications of our findings. These multifaceted considerations underscore the necessity of utilizing a blend of statistical methods in research and highlight the reliability and thoroughness of our results, providing valuable insights into the potential association between PM_2.5_ exposure and UC.

The penetration depth and deposition rate of particulate matter in the respiratory tract generally increase as the particle diameter decreases [22, 23]. During nasal inhalation, the nasal cavity’s cilia and mucus effectively filter particles larger than 10μm. These larger particulates predominantly settle in the upper respiratory tract, such as the nose and pharynx. Once inhaled, mechanisms like sneezing and coughing expel them [23]. Particles with diameters below 10μm are considered especially detrimental to health due to their ability to penetrate deeply into the respiratory tract, reaching the alveoli and potentially affecting the lower respiratory system [24]. Their presence can compromise respiratory health, increasing the risk of diseases like pneumonia and asthma. Additionally, these particles can adversely affect the gastrointestinal system, entering through ingestion of contaminated substances, inhalation, or respiratory secretions. They may also be transported to the oropharynx via absorption by alveolar macrophages and mucociliary actions [25]. Particulate matter’s harmful effects often arise from inducing cellular oxidative stress, leading to cytokine release and disrupting barrier functions [26]. Research by Mutlu E. A. et al. highlighted that intense particulate exposure can cause oxidative-dependent gastrointestinal epithelial cell death, damage tight junction proteins, induce inflammation, and elevate intestinal permeability. The barrier disruption is associated with reactive oxygen species (ROS) produced by these cells. Further studies demonstrated that such exposure significantly alters the microbial diversity in the small intestine, colon, and feces, with notable changes in bacterial composition, especially in the distal intestine, across all taxonomic levels. Moreover, particulate exposure was found to upregulate tumor necrosis factor-α (TNF-α) expression in the colon [19, 27]. These results suggest PM_2.5_ exposure in the intestines may contribute to inflammatory bowel diseases by impairing epithelial barriers and altering gut microbiota.

In summary, Mendelian randomization is a powerful tool for exploring the associations between exposures and outcomes, enabling clearer insights into causative relationships. Compared to randomized controlled trials, Mendelian randomization offers several advantages, such as addressing potential confounders, mitigating reverse causality, and optimizing the use of resources and time. To our knowledge, there are no Mendelian randomization studies linking airborne particulate matter with UC. One primary advantage of our research, compared to smaller-scale studies based on individual data, includes: 1. Leveraging genetic data from large-scale GWAS about airborne particulate matter and UC, enhancing the ability to identify causal relationships; 2. The broad distribution of genetic variations across chromosomes means that gene-gene interactions have minimal impact on results. However, our study has its limitations: 1. Since the genetic variations analyzed are derived solely from European cohorts, further studies are essential to validate the relevance of our findings to other populations and ethnicities; 2. The reliance on GWAS data limits our capacity to investigate potential non-linear relationships or variations arising from factors like age, health condition, or gender, which could contribute to the detected heterogeneity. Thus, more extensive Mendelian randomization studies or randomized controlled trials are recommended for future research to obtain more definitive conclusions.

## 5. Conclusion

Through Mendelian randomization, our study suggests a plausible causal link between PM_2.5_ exposure and UC onset. This finding indicates a potential avenue for preventive research. However, it is imperative to validate these associations in more expansive cohorts.

## Supporting information

S1 File Contains all the supporting tables. (XLSX)
